# Could platelet-to-lymphocyte ratio be a predictor for contrast-induced nephropathy in patients with acute coronary syndrome? A systematic review and meta-analysis

**DOI:** 10.1097/MD.0000000000016801

**Published:** 2019-08-09

**Authors:** Jie Jiang, Hong-Yan Ji, Wei-Ming Xie, Lu-Sen Ran, Yu-Si Chen, Cun-Tai Zhang, Xiao-Qing Quan

**Affiliations:** a Department of Geriatrics; b Second Clinical School, Tongji Hospital, Tongji Medical College, Huazhong University of Science and Technology, Wuhan, China.

**Keywords:** acute coronary syndrome, contrast-induced nephropathy, meta-analysis, platelet to lymphocyte ratio

## Abstract

**Background:**

Contrast-induced nephropathy (CIN) is acute renal failure observed after administration of iodinated contrast media during angiographic or other medical procedures. In recent years, many studies have focused on biomarkers that recognize CIN and/or predict its development in advance. One of the many biomarkers studied is the platelet-to-lymphocyte ratio (PLR). We performed a systematic review and meta-analysis to evaluate the correlation between PLR level and CIN.

**Methods:**

Relevant studies were searched in PUBMED, EMBASE, and Web of Science until September 15, 2018. Case-control studies reporting admission PLR levels in CIN and non-CIN group in patients with acute coronary syndrome (ACS) were included. The pooled weighted mean difference (WMD) and 95% confidence intervals (95%CI) were calculated to assess the association between PLR level and CIN using a random-effect model.

**Results:**

Six relevant studies involving a total of 10452 ACS patients (9720 non-CIN controls and 732 CIN patients) met our inclusion criteria. A meta-analysis of 6 case-control studies showed that PLR levels were significantly higher in CIN group than those in non-CIN group (WMD = 33.343, 95%CI = 18.863 to 47.823, *P* < .001, I^2^ = 88.0%).

**Conclusion:**

For patients with ACS after contrast administration, our meta-analysis shows that on-admission PLR levels in CIN group are significantly higher than those of non-CIN group. However, large and matched cohort studies are needed to validate these findings and assess whether there is a real connection or just an association.

## 1 Introduction

Contrast-induced nephropathy (CIN) is acute renal failure observed after administration of iodinated contrast media during angiographic or other medical procedures.^[1]^ CIN is reported to be the third most common cause of acute kidney injury in hospitalized patients, after ischemic and drug-induced injury.^[2]^ CIN is associated with mortality, cardiovascular events, renal failure, and prolonged hospital stay.^[3]^

Acute coronary syndrome (ACS), characterized by unstable atherosclerotic lesions, is the leading cause of death from coronary heart disease.^[4]^ CIN in patients with ACS is associated with short- and long-term adverse outcomes, including increased mortality and increased risk of ischemic and hemorrhagic events.^[5–7]^

Since CIN has been shown to be a potentially preventable clinical condition,^[8]^ there is an urgent need to identify factors that can predict the development of CIN. Although the pathogenesis and the underlying biological mechanisms of CIN have not yet been fully understood, it has been shown that inflammation may play an important role in the pathophysiology of CIN.^[9]^ At the same time, it is worth noting that high PLR reflects inflammation, atherosclerosis and platelet activation.^[10]^ In recent years, some studies have reported that CIN group had higher admission PLR levels compared with non-CIN group in patients with ACS after percutaneous coronary intervention (PCI) or angiography, suggesting that an increase in PLR may be a potential predictor of CIN.^[11–16]^

PLR has showed to be an inexpensive and convenient method for predicting the development of CIN in patients with ACS after PCI or angiography.^[11–16]^ However, in the current study, the outcomes were diverse.^[11–16]^ Therefore, we carried out a systematic review and meta-analysis to comprehensively evaluate the admission PLR levels in CIN group and non-CIN group in patients with ACS after PCI or angiography.

## 2 Methods

We adhered to the Preferred Reporting Items for Systematic Reviews and Meta-Analyses (PRISMA). Ethical approval was not necessary because our data was based on published articles.

### 2.1 Search strategy

We conducted a comprehensive computer search through the following databases from their inception until September 15, 2018: PubMed, EMBASE and Web of Science. The following terms were used in different combinations to identify relevant studies assessing the association between admission PLR and CIN: platelet-to-lymphocyte ratio, contrast medium, contrast media, contrast material, kidney diseases, nephropathy, acute kidney injury, acute renal injury, renal disease, CIN, contrast-associated nephropathy, contrast-induced acute kidney injury, radiographic contrast nephropathy, acute coronary syndrome, myocardial infarction, myocardial ischaemia, and unstable angina. Manual searching was performed for the reference lists of all included articles to identify additional eligible studies.

### 2.2 Inclusion and exclusion criteria

Studies that satisfied the following criteria were included:

(1) case-control study;
(2) clear definition and diagnosis of CIN for human participants;
(3) reporting mean and standard deviation (SD) values or median and interquartile range of admission PLR levels for cases and controls;
(4) The study population is patients with ACS.

We excluded review articles, conference articles, animal studies, and other irrelevant clinical trials. In cases of similar articles published by the same team, we examined the data to determine whether it had come from the same study. If it had, only the article with a larger sample size or more accurate data was included.

### 2.3 Data extraction and quality assessment

Two investigators independently extracted data from all of the included studies using a standardized data collection form for analysis. Disagreements were resolved by discussion between the 2 investigators. The following information on study characteristics was extracted from each article: the first author's name, publication year, country, study population, sample size, baseline characteristics of cases and controls (age, male, hypertension, admission glucose, hemoglobin, baseline serum creatinine), the definition of CIN, study design, mean and standard deviation (SD) or median and interquartile range of admission PLR levels for CIN and non-CIN group.

The quality of studies was assessed by the Newcastle–Ottawa Scale (NOS), which is specifically developed to evaluate the quality of nonrandomized observational studies.^[17,18]^ The NOS ranges from 0 to 9 stars. The categories included high quality (score 7–9), medium quality (score 4–6) and low quality (score < 4).^[19]^

### 2.4 Statistical analyses

All statistical analyses were performed using STATA version 12.0 (STATA, College Station, TX) and Review Manager (RevMan 5.3, Cochrane Collaboration, Nordic Cochrane Center, Copenhagen, Denmark).

If the quantitative data is given as median and interquartile range, mean values were estimated using the method as described previously.^[20]^ SD values were estimated using the method as described previously.^[21]^ Effect sizes were expressed as the weighted mean difference (WMD) and their 95% confidence intervals (95%CI). A *P* value of .1 or less was considered statistically significant.

Heterogeneity between the results of different studies was examined by χ^2^ tests for significance (a *P* value <.1 was considered statistically significant) and I^2^ test.^[22]^ We regarded an I^2^ value of <25%, 25% to 50%, and > 50% as low, moderate, and high amounts of heterogeneity, respectively.^[22]^ A *P* value >.1 and I^2^ value of <50% were considered to be of no significant heterogeneity, and a fixed-effect model was used.^[23,24]^ Otherwise, use a random-effect model.^[25]^

Subgroup analyses were performed by sample size, study population, geographic locations and the definition of CIN. A meta-regression analysis of single covariate was performed by a Restricted Maximum Likelihood (REML) random effect when studies showed high heterogeneity. WMD was used as the dependent variable. Age, male (%), hypertension (%), admission glucose, hemoglobin and baseline serum creatinine of cases were entered as explanatory covariates. A *P* value <.1 was considered statistically significant.

Publication bias was evaluated using Egger test. A *P* value <.1 was considered statistically significant. Influence analysis was undertaken by omitting one study at a time to examine influence of one study on the overall summary estimate.^[26]^

## 3 Results

### 3.1 Study selection

**Figure 1:**
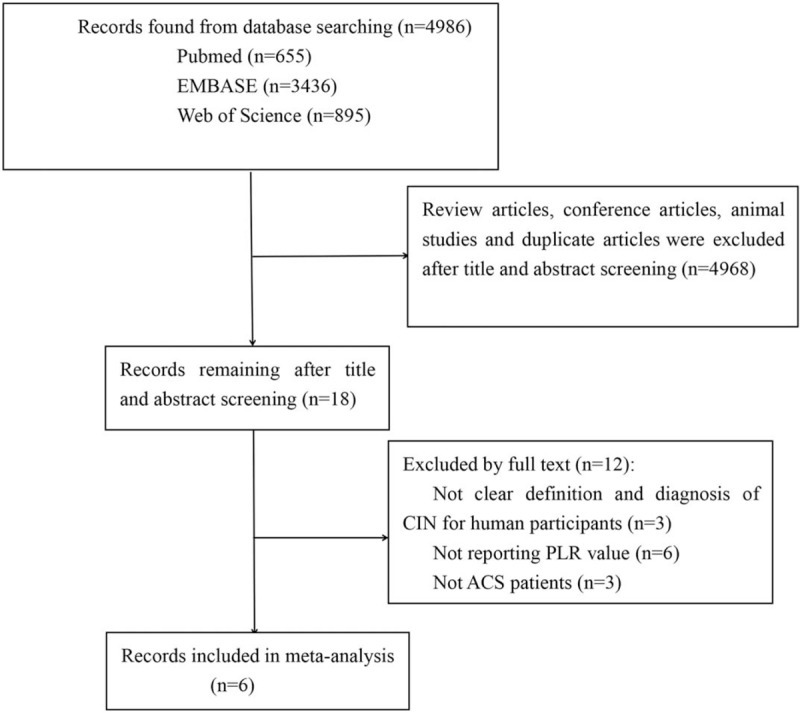
The process of study selection for the meta-analysis.

In all, 4986 articles were identified from the primary literature search. After screening of the titles and abstracts, review articles, meeting abstracts, animal studies, and duplicate articles were excluded. Eighteen articles were identified. Of these, 12 articles were excluded after full-length paper evaluation, leaving 6 studies for meta-analysis. The detailed search strategy was shown in the flow diagram (Fig. 1).

### 3.2 Study characteristics

We identified 6 case-control studies that reported the relationship between PLR levels and CIN in patients with ACS after PCI or angiography. The 6 studies were published between 2015 and 2018 and reported data on 10,452 subjects (9720 non-CIN controls and 732 CIN patients). All subjects were selected randomly without sex restriction. The definition of CIN was different among included studies. The main characteristics of included studies were summarized in Table 1. And additional baseline clinical data of included studies were reported in Table 2. In addition, quality assessment scores ranged from 7 stars to 8 stars. NOS quality assessment results were reported in Table 3.

**Table 1:**
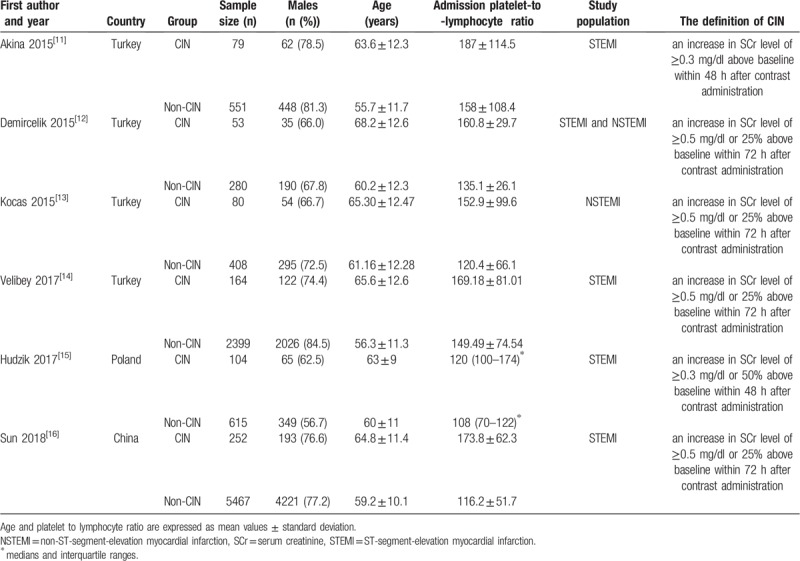
Main characteristics of included studies.

**Table 2:**
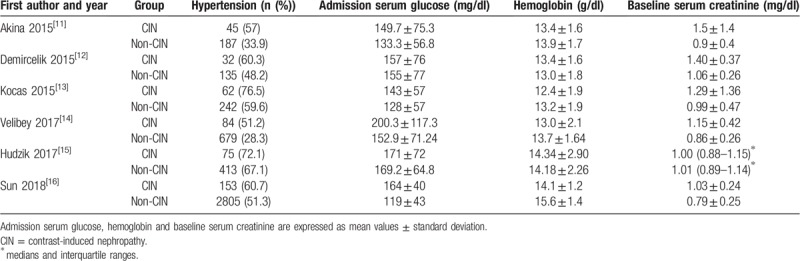
Additional baseline clinical data of included studies.

**Table 3:**
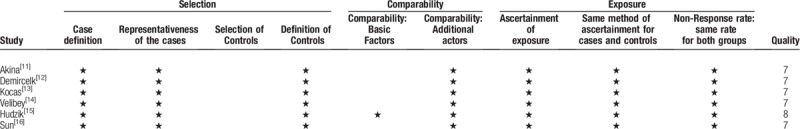
Newcastle-Ottawa Scale.

### 3.3 PLR levels and CIN

**Figure 2:**
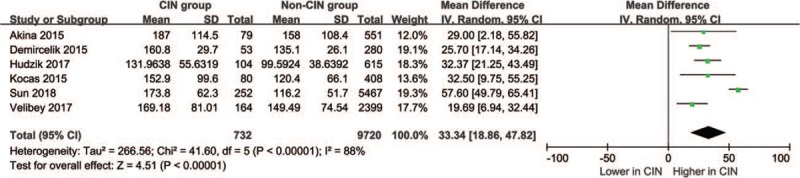
Association between admission PLR levels and CIN in patients with ACS. ACS = acute coronary syndrome, CIN = contrast-induced nephropathy, PLR = platelet-to-lymphocyte ratio.

Overall, 10,452 participants were included in the 6 studies. Because there was a significant heterogeneity (I^2^ = 88.0%, *P*_heterogeneity_ <.001) across 6 studies, we selected the random-effect model for analyses. A meta-analysis of 6 case-control studies showed that PLR levels were significantly higher in CIN group than those in non-CIN group in patients with ACS (WMD = 33.343, 95%CI = 18.863 to 47.823, *P* < .001). Combined analysis of the relationship between the PLR levels and CIN was shown in forest plot (Fig. 2).

### 3.4 Meta-regression analysis and subgroup analysis

There was significant heterogeneity among these studies (I^2^ = 88.0%, *P*_heterogeneity_ < .001). Therefore, we conducted meta-regression analysis and subgroup analysis to explore the source of heterogeneity.

**Figure 3:**
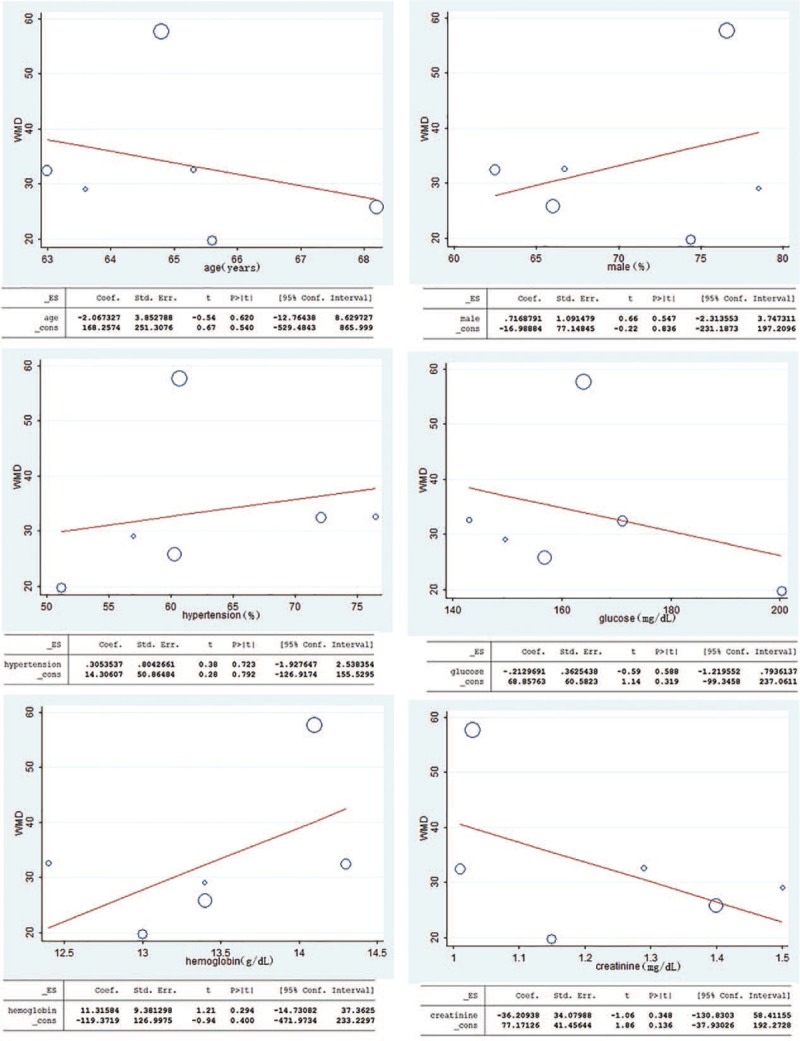
Meta-regression analysis assessing the impact of age, male (%), hypertension (%), admission glucose, hemoglobin and baseline serum creatinine on WMD. WMD = weighted mean difference.

To investigate the impact of the predefined study-level characteristics on WMD in PLR, REML-based random effect meta-regression analysis was performed. WMD was used as the dependent variable. Age, male (%), hypertension (%), admission glucose, hemoglobin and baseline serum creatinine of cases were entered as explanatory covariates. *P* values of single covariate meta-regression analysis with the covariates of age, male (%), hypertension (%), admission glucose, hemoglobin and baseline serum creatinine were .620, .547, .723, .588, .294, and .348, respectively. The results showed that no covariates had a significant impact on between-study heterogeneity. The meta-regression analysis detailed results were reported in Figure 3.

Subgroup analyses based on the characteristics of participants were introduced to explore the potential source of heterogeneity. We performed predefined subgroup analyses for sample size, study population, geographic locations and the definition of CIN. In the subgroup with sample size less than 1000 and Europe, there was no significant heterogeneity among the studies (Table 4). Therefore, the sample size and geographic locations were possibly the origins of the significant heterogeneity of the pooled data in our meta-analysis.

**Table 4:**
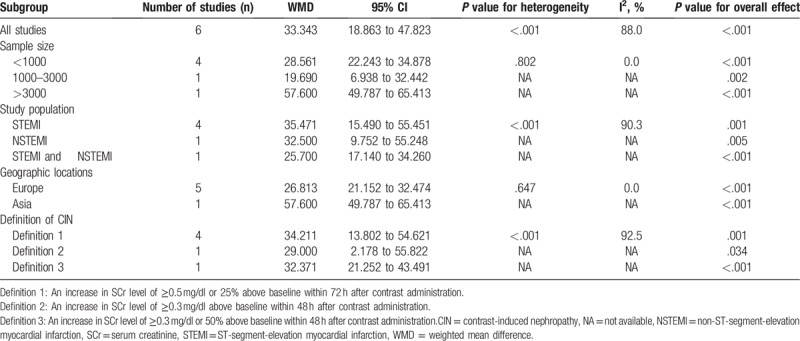
Summary WMD for overall and subgroup results.

### 3.5 Publication bias

**Figure 4:**
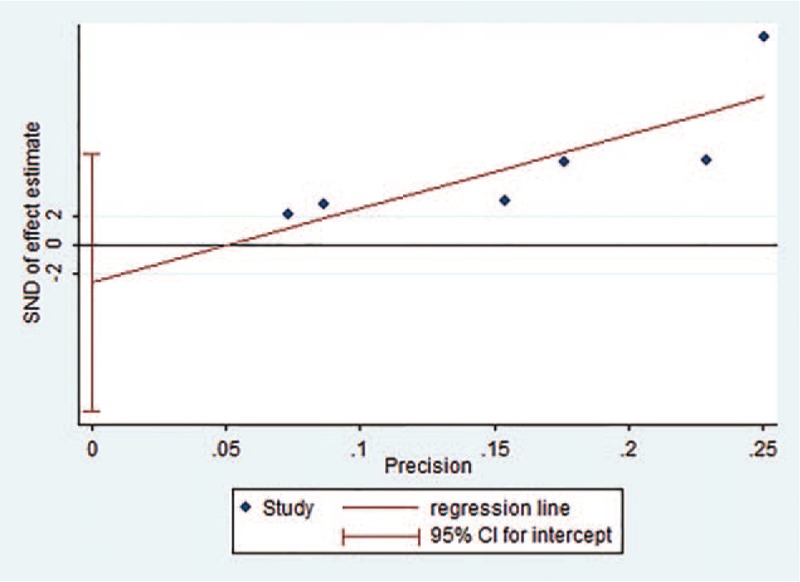
Egger publication bias plot. SND = standard normal deviate.

Publication bias was assessed using Egger test in the meta-analysis. There was no evidence of publication bias according to the results of Egger linear regression (intercept = −2.62, 95%CI = −11.54 to 6.30, *P* = .460), which suggested low risk of publication bias in the meta-analysis. Egger publication bias plot was shown in Figure 4.

### 3.6 Influence analysis

**Figure 5:**
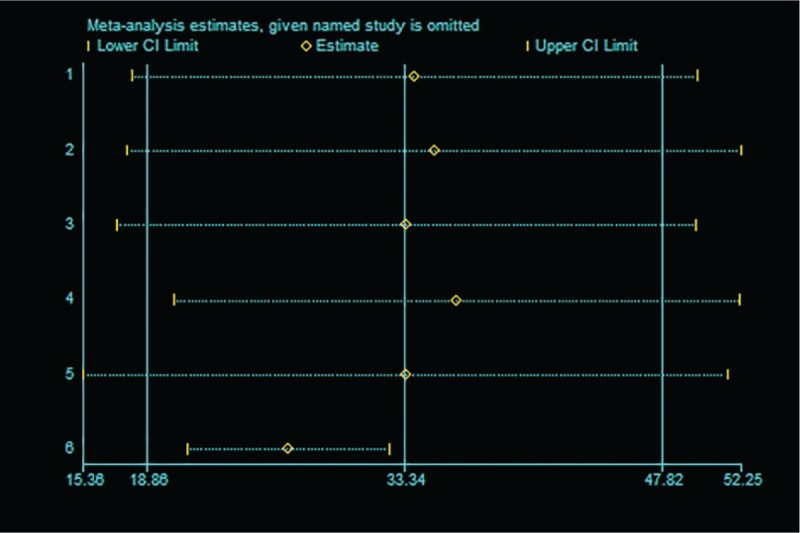
Influence analysis of individual study on the pooled estimate for studies on the association between admission PLR levels and CIN. The pooled WMD is reestimated after omitting one study (the “named study” in the left of the graph) each time; the circle in each horizontal line represents the pooled WMD, and the length of the short dash line represents the 95% confidence interval (CI) of the pooled WMD. CIN = contrast-induced nephropathy, PLR = platelet-to-lymphocyte ratio, WMD = weighted mean difference.

To determine the influence of each study on the overall result, the stability of results was evaluated using a leave-one-out strategy. This method excludes each individual study to determine whether there is a significant change in the pooled values. In the influence analysis, after excluding each individual study the mean difference ranged from 26.81 (95%CI = 21.15 to 32.47) to 36.28 (95%CI = 20.40 to 52.16). The graph of influence analysis was shown in Figure 5. After excluding the study of Sun et al,^[16]^ which reported highest mean difference between the 2 groups, the PLR levels remained significantly higher in CIN patients than non-CIN controls (WMD = 26.81, 95%CI = 21.15 to 32.47).

## 4 Discussion

In recent years, PLR studies started a new field for CIN research and there were studies showing PLR might be an independent predictor of CIN in patients with ACS after PCI or angiography.^[11–16]^ The present meta-analysis was conducted in 6 studies, including 9720 non-CIN controls and 732 CIN patients. To the best of our knowledge, this was the first meta-analysis to evaluate the relationship between the admission PLR levels and CIN in patients with ACS after PCI or angiography. The main finding of the present study was that the PLR levels in the CIN group were higher than those in the non-CIN group (WMD = 33.343, 95%CI = 18.863 to 47.823, *P* < .001).

CIN has been known as contrast-induced acute kidney injury and is a major adverse effect caused by exposure to intravascular iodinated contrast medium.^[27]^ The exact pathophysiology of CIN is still not well known, but several factors have been strongly implicated to have some roles in its development, including intrarenal vasoconstriction, reduced renal blood flow, oxidative stress, inflammation, renal ischemia, reactive oxygen species formation, reduction of nitric oxide production, tubular epithelial, and vascular endothelial injury.^[1,28]^

Currently, there are several potential risk factors identified for CIN, including diabetes mellitus; hyperuricemia; multiple iodinated contrast media doses within a short time (<24 h); advanced age; the amount and type of the contrast medium as well as the type of the intervention for which contrast media is used.^[1]^ In patients without risk factors, the incidence of CIN appears to be small (<1%).^[29]^ But in high-risk patients the incidence appears to be high (up to 15%).^[30]^ Because there is evidence that CIN is preventable,^[8]^ it is important to identify high-risk patients to take steps in advance to reduce the incidence of CIN.

On the one hand, higher platelet count was an independent risk factor for CIN in patients with diabetes or baseline kidney dysfunction.^[31]^ On the other hand, in patients with non-ST-segment-elevation myocardial infarction (NSTEMI) after PCI, lymphocyte counts were significantly lower in the CIN group compared with the non-CIN group.^[32]^ What is more, previous studies have shown that PLR is an independent predictor of CIN in ACS patients after PCI or angiography.^[11–16]^ Akin, Velibey, and Sun found that the PLR levels of the CIN group were significantly higher than those of the non-CIN group in patients with ST-segment elevation myocardial infarction (STEMI) who underwent primary PCI.^[11,14,16]^ Hudzik also found similar relationship in patients with diabetes and STEMI.^[15]^ Demircelik and Kocas also found CIN had higher admission PLR levels compared with non-CIN in patients with NSTEMI.^[12,13]^ The advantage of PLR is that it reflects both overactive coagulation and inflammatory pathways, which are the underlying mechanisms of CIN, suggesting that PLR may be a valuable marker for CIN.^[13]^

When analyzing the heterogeneity of the included literature, we found significant heterogeneity (I^2^ = 88.0%, *P*_heterogeneity_ < .001). Thus, we performed meta-regression and subgroup analysis to explore the possible causes of heterogeneity. In the meta-regression, WMD was used as the dependent variable. Age, male (%), hypertension (%), admission glucose, hemoglobin and baseline serum creatinine of cases were entered as explanatory covariates. However, no covariates had a significant impact on between-study heterogeneity. Then, subgroup analyses by the sample size, study population, geographic locations and the definition of CIN were performed to explore the source of heterogeneity. In the subgroup with sample size less than 1000 and Europe, there was no significant heterogeneity among the studies (Table 4), suggesting that heterogeneity among those studies might arise from geographic locations and sample size.

As a meta-analysis of published studies, our findings showed some advantages. First, this is the first comprehensive meta-analysis to study the association between PLR levels and CIN. Second, a large number of participants were included (9720 non-CIN controls and 732 CIN patients), allowing a much greater possibility of reaching reasonable conclusions between PLR levels and CIN. Third, in the influence analysis, the overall results and conclusions were not significantly affected after the deletion of any of the studies, suggesting that the meta-analysis results were more credible.

Despite these meaningful findings, some limitations should be noticed. First, the studies included in the meta-analysis were case-control studies. Case-control studies have their own limitations. On the one hand, case-control studies do not confirm the causal relationship between exposure and disease. On the other hand, case-control studies have selective bias and recall bias. However, the papers we can find and can be used for meta-analysis on the relationship between PLR and CIN are all case-control studies, so the included studies are all case-control studies, which made the results of our meta-analysis not as reliable as the results of the meta-analysis including randomized controlled trials. Therefore, randomized controlled studies in this area are needed in the future. Second, significant heterogeneity was found in this meta-analysis, but the heterogeneity among those studies was not fully explained by the subgroup analysis and meta-regression. On the one hand, we found that the European group did not have significant heterogeneity, so speculation that heterogeneity might be derived from geography. On the other hand, because five of the included studies were from Europe and only one was from Asia, the lack of data from the Asian group made the conclusion that heterogeneity originated from geography was insufficient. More data from Asia is needed to make this conclusion more reliable. Third, the number of the included studies was relatively small, which limited the reliability of meta-analysis results to some extent. More large well-designed studies are required to confirm the associations between PLR levels and CIN. Fourth, a total of 6 studies were included, but five of them were from Europe and only one from Asia. Due to this limitation, the results may be more applicable to Europe. More studies originating in other countries are required to investigate the association between PLR levels and CIN. Fifth, these results should not be extrapolated to all patients with ACS who underwent coronary angiography or PCI because patients with unstable angina were not included in all included studies.

In addition to predicting contrast-induced nephropathy, there are studies on the use of contrast-free techniques to prevent contrast-induced nephropathy. Direct delineation of myocardial infarction from non-contrast agents cardiac magnetic resonance imaging sequences using a joint motion feature learning architecture was reported.^[33]^ This technique can be used for monitoring and follow-up in patients with myocardial infarction. In addition, the use of chlorophyll derivatives emissions as biomarkers was also reported.^[34]^ Chlorophylls and chlorophyll derivatives show bright emission bands at long-wavelength regions (∼675 and ∼720 nm). This technique can monitor bowel perforation in real time in the surgical setting without synthetic contrast agents. In addition to the above, Advance Practice Provider utilizing a pre-catheterization screening tool decreased patients’ risk of CIN.^[35]^

## 5 Conclusions

For ACS patients after angiography or PCI, admission PLR levels are significantly higher in CIN group compared to non-CIN group. Therefore, PLR could be a potential predictor for CIN in patients with ACS after angiography or PCI. However, large and matched cohort studies are needed to validate these findings and assess whether there is a real connection or just an association.

## Acknowledgments

We thank all participating investigators for providing patients’ data for the present meta-analysis.

## Author contributions

**Conceptualization:** Jie Jiang, Hong-Yan Ji, Wei-Ming Xie, Lu-Sen Ran, Yu-Si Chen, Cun-Tai Zhang, Xiao-Qing Quan.

**Data curation:** Jie Jiang, Xiao-Qing Quan.

**Formal analysis:** Jie Jiang, Xiao-Qing Quan.

**Investigation:** Jie Jiang, Xiao-Qing Quan.

**Methodology:** Jie Jiang, Xiao-Qing Quan.

**Project administration:** Jie Jiang.

**Resources:** Jie Jiang.

**Software:** Jie Jiang.

**Supervision:** Jie Jiang.

**Validation:** Jie Jiang.

**Visualization:** Jie Jiang.

**Writing – original draft:** Jie Jiang, Xiao-Qing Quan.

**Writing – review & editing:** Jie Jiang, Hong-Yan Ji, Wei-Ming Xie, Lu-Sen Ran, Yu-Si Chen, Cun-Tai Zhang, Xiao-Qing Quan.
